# Profile of adults users of smartphone applications for monitoring the level of physical activity and associated factors: A cross-sectional study

**DOI:** 10.3389/fpubh.2022.966470

**Published:** 2022-09-20

**Authors:** Wesley de Oliveira Vieira, Thatiane Lopes Valentim di Paschoale Ostolin, Maria do Socorro Morais Pereira Simões, Neli Leite Proença, Victor Zuniga Dourado

**Affiliations:** ^1^Laboratory of Epidemiology and Human Movement, Department of Human Movement Sciences, Institute of Health and Society, Federal University of São Paulo (UNIFESP), São Paulo, Brazil; ^2^Lown Scholars Program, Harvard T.H. Chan School of Public Health, Boston, MA, United States

**Keywords:** mobile Health (mHealth), Brazil, physical fitness, risk factors, demographics

## Abstract

**Background:**

There are currently more than 200 million smartphones in Brazil. The potential of mobile technologies for favorable changes in health behavior such as physical activity has been previously described in the literature. Results of surveys in developed countries indicate that applications (APPs) are developed for people who are better educated, younger, and with higher incomes compared to non-users. However, the profile of users in developing countries like Brazil is not well-known. Understanding the profile of APP users might ease the development turned to physically inactive people and those at higher cardiovascular risk. Furthermore, the physiological and functional factors associated with the use of such APP are unknown.

**Objectives:**

To characterize the profile of APP users to monitor the physical activity level (PAL) and assess the demographic, socioeconomic, clinical, physiological, and functional characteristics associated with the use of smartphone APPs to monitor physical activity in Brazilian adults.

**Methods:**

We assessed 176 asymptomatic men and 178 women (43 ± 12 years; 27 ± 5 kg/m^2^). We initially asked participants about their current use of a smartphone APP containing PAL monitoring functionality, such as exercise session logs and/or step counts. In a cross-sectional design, we investigated schooling, socioeconomic status (*Critério Brasil*), and classic self-reported cardiovascular risk factors. We evaluated several physiological and functional variables such as maximum O_2_ consumption on a treadmill (VO_2_ max), blood pressure, body composition (bioelectrical impedance), handgrip strength, and isokinetic muscle strength of the dominant lower limb. Participants used a triaxial accelerometer for 7 days to quantify daily physical activity. We also assessed health-related quality of life (WHOQOL BREF), perceived stress (PSS14 Scale), and the built environment (NEWS Scale). We compared continuous variables using the Student's *t*-test and categorical variables using the χ^2^ test, between APP users and non-users. After univariate analysis, we included the main variables associated with the use of APP in a multiple logistic regression model.

**Results:**

One hundred and two participants (28.3%), unrelated to gender, reported using a smartphone APP for physical activity at the time of assessment. Except for perceived stress and the built environment that were not associated with the use of APP, users of APP were younger and had higher education, lower cardiovascular risk, better socioeconomic status, a better quality of life, better cardiorespiratory function, better body composition, greater physical fitness and more moderate to vigorous physical activity in daily life. The results of the multiple logistic regression showed that age, hypertension, VO_2_ max, socioeconomic status (*Critério Brasil*), and quality of life (WHOQOL BREF total score) were the variables most significantly associated with the use of the APP.

**Conclusions:**

Our results indicate that smartphone APPs to monitor physical activity are developed for younger adults with better socioeconomic status, lower cardiovascular risk, higher quality of life, and greater cardiorespiratory fitness. Greater efforts are needed to develop a science-based APP for people who most need this technology, enabling greater potential to prevent undesirable health outcomes in asymptomatic adults.

## Introduction

The use of smartphone applications (APP) that encourage good health habits and monitor the level of physical activity in daily life (PAL) has increased in recent years (1). Its use is associated with the adoption of good health habits, as well as an increase in PAL (e.g., number of steps and caloric expenditure) (1–5).

In developed countries, it was reported that APP users are more educated, younger, and have higher income and PAL compared to non-users (1, 2, 4). Even with this information, most APPs currently available for download are not developed based on scientific evidence (6, 7). This fact can discourage new people from accessing this technology and limit its effectiveness in increasing the PAL of those who already use it (6). The growth in the use of this type of APP also occurred in developing countries (8). Despite this, little is known about the profile of this public in these places (8). To our knowledge, no study aiming to collect these data has been conducted in Brazil.

In Brazil, despite the economic crisis, the demand for smartphones has increased dramatically. According to a survey carried out by Getúlio Vargas Foundation, there are 346 million portable devices in use in Brazil (notebooks, tablets, and smartphones), with 242 million smartphones totaling 114% per capita (9).

Certainly, there is great potential for smartphones to improve cardiovascular health in Brazil. Despite the impressive number of smartphones in Brazil, there is no information about the usability of the APP to increase PAL. Family income and educational level are determinants for the lower use of such APPs (10), even so, a study confirmed the usability of a physical activity APP in a rural area of the United States (11). Although it is limited in quantity and quality, there is evidence of the effectiveness of mobile devices in low- and middle-income countries (12).

Among the mechanisms by which APPs could contribute to increases in the level of physical activity is the possibility to offer a combination of behavior change techniques in one device (13, 14). Pontin et al. (15) investigated socio-demographic determinants of physical activity among APP users and showed that most were female, aged from 25 to 50 years old. The authors highlight that the availability of more demographic and location information from APP users would contribute to studies using smartphone APP.

Knowing this, it is clear the importance of obtaining information about the profile of this public. Based on current evidence, we hypothesized that APP users who monitor the PAL have the same sociodemographic characteristics mentioned above in developed countries. However, factors inherent to developing countries like Brazil, such as urban violence, the scarcity of built environments for physical activity, as well as psychosocial stress can negatively impact the willingness to use smartphone APPs in physical activities outdoors. The determining factors for the use of these APPs in Brazil were not investigated, especially the last three mentioned above.

In this sense, sociocultural determinants must be considered to scientifically support such APPs for the use of Brazilians and to promote the cardiovascular health of users. Through this information, we will provide the first view of APP users and non-users in a sample of Brazilians. This can help programmers in creating new APPs that are more personalized, attractive to Brazilians, and effective in increasing PAL about existing ones. Therefore, the objective of this study is to characterize the profile of APP users to monitor PAL and to evaluate the demographic, socioeconomic, clinical, physiological, and functional characteristics associated with the use of smartphone APPs to monitor PAL in adults residing in *Baixada Santista* region.

## Methods

### Study design, setting, and ethics

This is a cross-sectional study, conducted at the Laboratory of Epidemiology and Human Movement of UNIFESP, located in Santos, São Paulo, Brazil. The study was approved by the Ethics Committee in Research with Human Beings of the Federal University of São Paulo (UNIFESP - n° 0676/2018), and all volunteers signed the informed consent before participation.

### Study sample

We invited volunteers through disclosures made on social networks and pamphlets posted at the laboratory's host institution, constituting a convenience sample. They were instructed to call the telephone center to schedule the evaluations, where we informed them about the inclusion criteria of the study and its potential risks and benefits.

We included in the study people aged 18 or over, residents of the *Baixada Santista* region, literate and free from musculoskeletal, cardiorespiratory, and/or cognitive impairments that could interfere with the correct execution of the assessments.

We excluded from the study those who did not complete or refused to perform all assessments, those who did not have valid accelerometry, who did not have a smartphone or did not take it, as well as those who were detected by our team with any risk of adverse cardiovascular events through exercise test (severe obstructive ventilatory disorder, severe arrhythmias, or stable angina during exercise testing or at rest and ST-segment depression).

### Sample size calculation

The sample required for this study was calculated based on the sociodemographic, cultural, and functional characteristics of smartphone APP users who implement PAL monitoring. For this, we used the multiple regression sample calculation tool www.statstodo.com. To investigate the main attributes that determine the use of APP, we selected the 10 variables with the highest association after univariate analysis (i.e., with *p* < 0.1). Considering an alpha error probability of 5%, statistical power of 80%, a correlation coefficient multiple of 0.30 (e.g., low effect size), and up to 10 predictors in the multiple logistic regression model, 173 individuals would be sufficient to answer our question of research.

We considered as potential predictor variables for the use of APP the following data: age, sex, socioeconomic status, hypertension, smoking, diabetes, dyslipidemia, fat percentage, stages of behavior for physical exercise, quality of life, maximum oxygen consumption (VO_2_ max), number of steps, time of moderate to vigorous physical activity (MVPA), handgrip strength and peak torque of knee extension. We also consider the built environment and perceived stress in the multiple models.

### Data collection procedures

The evaluations were carried out on 2 different days, respecting 1 week between them. On the first day, we performed, in the following order, the evaluations of demographic data, clinical history, socioeconomic status, behavioral stages for physical exercise, quality of life, perceived stress, use of APPs to monitor PAL, and cardiorespiratory fitness. After the evaluations, we delivered a triaxial accelerometer to the volunteer, instructing him/her on its use, as well as informing him/her about the recommendations to be followed for the second day of the study. The second day was used to return the accelerometer and perform the following assessments: anthropometry, body composition, handgrip strength, isokinetic muscle function, and perception of the built environment.

All assessments were performed by trained personnel, and all types of equipment were calibrated according to the manufacturer's instructions.

### Variables

#### Demographic data

We obtained, from the self-report of the volunteers, age (in years), sex (male or female), race (white, black, brown, native Indian, or yellow), and level of education (complete/incomplete high school).

#### Anthropometry

We measured the height (cm) and body mass (kg). For this, we used a digital scale with a stadiometer (Toledo®, São Paulo, Brazil). With these data, we calculated the body mass index (BMI = body mass in kg/height^2^). Anthropometric measurements were performed on the first day of assessments, and body mass and height data were used only for cardiorespiratory fitness assessment. Body mass, height, and BMI used in the results were obtained on the second day of evaluations. We did this because the body composition assessment was also performed on this day.

#### Socioeconomic status

We evaluated using the *Critério Brasil* questionnaire (16). Of the instruments for socioeconomic assessment available in Brazil, this is considered the most accurate and that is why it was used (17). The interviewer asked all 15 questions to the subject. The questions are subdivided into three groups: comfort items, public services, and the level of education of the person who contributes most to the family income (“head of the family”). We considered the total score obtained for the classification of socioeconomic status. The questionnaire also provides a socioeconomic classification of average monthly family income according to the score obtained: A (45 to 100 points/R$20.888), B1 (38 to 44 points/R$9.254), B2 (29 to 37 points/R$4.852), C1 (23 to 28 points/R$2.705), C2 (17 to 22 points/R$1.625), D–E (0 to 16 points/R$768) (16).

#### Clinical assessment

Initially, the participants answered the physical activity readiness questionnaire (PAR-Q) (18). Subsequently, we stratified the risk for cardiovascular events during exercise according to the ACSM recommendations (19). An interview was conducted based on the following cardiovascular risk factors: age (men > 45 years and women > 55 years), family history of cardiovascular diseases (acute myocardial infarction, revascularization, or sudden death before age 55 years for father or other relative first-degree male or before age 65 for mother or other female first-degree relatives), smoking (previous, current, or never), high blood pressure, dyslipidemia, diabetes mellitus, obesity, and physical inactivity [self-reported: <30 min of physical activity, continuously or cumulatively, for at least 5 days a week, with moderate to vigorous intensity (20)].

#### Stages of behavior change for the practice of physical exercise

According to the trans-theoretical model (21), we identified the stages of behavior change for the practice of physical exercise from the algorithm originally proposed by Cardinal et al. (22) and, according to Guedes et al. (23), validated and adapted to the Portuguese language. Based on their responses, all participants were classified in one of the following stages of behavior change proposed by the algorithm: maintenance, action, preparation, contemplation, and pre-contemplation. The regularly active lifestyle was designated through internationally proposed recommendations: performing at least 30 minutes of physical activity, either continuously or cumulatively, for at least 5 days a week, with moderate to vigorous intensity (20).

#### Quality of life

We used the Brazilian version of the WHOQOL BREF questionnaire (24). It was self-completed by the volunteers, and they were free to clarify possible doubts with the evaluators. This instrument consists of 26 questions. The first two represent, respectively, a general perception of quality of life and satisfaction with health. The answers follow a Likert scale from 1 to 5 (the higher the score, the better the quality of life). Excluding the first two questions, the questionnaire has 24 facets that make up 4 domains, namely: physical, psychological, social relationships, and environment. The result was presented through the arithmetic mean of the general score of the questionnaire. To calculate the mean, the scores for questions 3, 4, and 26 were inverted. We used the following classification for the result: needs improvement (1 to 2.9), regular (3 to 3.9), good (4 to 4.9), and very good (5) (24).

#### Perceived stress

We evaluated through the Brazilian version of the Perceived Stress Scale 14, which was validated, translated, and adapted by Luft et al. (25). This version was initially developed for the elderly, but it has also been validated for other adult populations in Brazil (26, 27). It consists of 14 questions, which were answered on a five-point Likert scale (0 = never; 1 = almost never; 2 = sometimes; 3 = almost always; 4 = always). The final score can range from 0 to 56 points. We followed the division of factors according to the original version of the scale (28). It indicates seven questions that make up a negative factor (1, 2, 3, 8, 11, 12, and 14) and seven questions related to the positive factor (4, 5, 6, 7, 9, 10, and 13). To calculate the final score, the positive factor questions had their scores inverted. To characterize the perceived stress level of the sample, we used the general cutoff score proposed by Faro (27) (≤ 18 = low; 19–24 = normal; 25–29 = moderate; 30–35 = high; and >35 = very high).

#### Cardiorespiratory fitness

We assessed VO_2_ max using a treadmill ramp protocol (ATL, Inbrasport, Curitiba, Brazil). After 3 min of walking comfortably, speed and incline were automatically increased according to the estimated maximum oxygen consumption to complete the test in about 10 min. The estimated VO_2_ max was calculated according to the ACSM recommendations (20). We monitored heart rate throughout the test using a 13-lead electrocardiogram (c12x, Cosmed, Pavona di Albano, Italy). Gas exchange and ventilatory variables were analyzed breath by breath, using a periodically calibrated computerized system for cardiorespiratory exercise testing (Quark PFT, Cosmed, Pavona di Albano, Italy). We used the following criteria to determine maximal effort: maximum heart rate of at least 90% of that predicted for age (220—age), gas exchange rate > 1.20, or VO_2_ plateau. In cases where these criteria were not met, the test would be repeated within a week. We obtain pulmonary oxygen uptake (VO_2_ peak) through the analysis of expired gases. The data were filtered every 15 s and the arithmetic mean of the VO_2_ obtained in the last 15 s at the peak of the incremental exercise was used as a representative of the VO_2_ peak of each participant.

#### Direct weekly PAL assessment

We used previously validated triaxial accelerometry (model G3TX, ActiGraph, MTI, Pensacola, FL) (29–31). The volunteers were instructed to wear the device on their hip on the dominant side of the body, attached to an elastic belt, within 1 week of the first day of testing in our study. We recommended that the device be removed to perform activities involving water, contact sports (fights, team and/or individual sports that involve falls, projections, or other types of physical shock that could damage the device), and during night sleep. Data were considered valid only when volunteers used the accelerometer for at least 4 days. Ten hours of use was all it took for 1 day to validate (29). In this validation period, at least 1 weekend day should be accounted for. The MVPA time was quantified according to the counts/minute threshold that was established in > 1951 (32). We also recorded the average number of steps (ANS) taken during the evaluation period. This data was also used to characterize the participants' PAL. For ANS, we considered the following classifications: ≤ 5,000 steps (sedentary), 5,001 to 9,999 (insufficiently active), and ≥10,000 steps (physically active) (33).

#### Body composition

It was evaluated by bioelectrical impedance (310e Biodynamics, Detroit, USA) performed at room temperature. All volunteers had fasted for at least 4 h and followed the other recommendations before performing the bioimpedance (34). We inform all of them in advance on the first day of evaluations. Resistance and reactance were determined with the volunteer in the supine position, with arms and legs abducted at 30 and 45°, respectively (34, 35). After cleaning the region with cotton wool soaked in alcohol, we placed two electrodes on the hand and foot on the dominant side. Lean body mass (kg) and body fat percentage were calculated using the group-specific regression equation developed by Kyle et al. (34) for healthy individuals. After the evaluation, we allowed the volunteers to eat and hydrate *ad libidum*.

#### Isokinetic muscle function

Muscle strength was considered another component of physical fitness included in the study. For this, we evaluated the muscle function of the knee joint using isokinetic dynamometry (Biodex System 3, Lumex Inc., Ronkonkoma, NY, USA), according to the procedures described by Malaguti et al. (36). The volunteers remained seated with the trunk and the dominant lower limb completely fixed by velcro straps. The knee joint assessment piece was attached to the mechanical axis of the dynamometer. We aligned the axis of rotation of the evaluated joint to the axis of rotation of the device. The ankle was supported and fixed on the upholstery of the piece, which was positioned above the lateral malleolus. After a session of 3 to 5 repetitions for warm-up and familiarization with the test, the peak knee extension torque (PT, Nm) was evaluated in five movements of extension and flexion, in concentric contraction, at 60°/s. During the execution of the test, we encouraged the volunteers, through strong verbal encouragement, to perform at their best. We recorded the highest PT value obtained for analysis.

#### Handgrip strength

We assessed the handgrip strength of the dominant hand using a hydraulic dynamometer (SH5001 SAEHAN®) as recommended (37). We define the dominant hand as the hand that is used frequently to carry out activities of daily living. We positioned the volunteers seated in a chair, with the arm adducted, the elbow flexed at 90°, and the forearm in a neutral position. Wrist hyperextension of up to 30° and ulnar deviation of up to 15° was allowed during the test. Each participant performed three tests with an interval of 30 s between them. We considered the highest value obtained for the statistical analysis.

#### Built environment

We evaluated the perception of elements of the community environment related to physical activity. To this end, we used the Brazilian version of the neighborhood environment walkability scale (NEWS) developed by Saelens et al. (38) and adapted and validated for Brazil by Malavasi et al. (39). NEWS included 83 questions divided into 9 items. Each item addressed the following themes: residential density, proximity to stores and businesses in general, perception of access to these places, street characteristics, walking and cycling facilities, neighborhood surroundings, and safety about traffic and crime. We scored the questions on a scale of 1 to 4, except for the first two and the last item, considering the ones mentioned above. In these questions, we used a scale from 1 to 5, and in the second item, the questions have an extra alternative for those who could not answer, characterized by the number 8. This extra question had the same score as the answer 5. This fact occurred for the reason mentioned forward. The second item of the questionnaire addressed the time spent walking to reach certain commercial establishments. Answer five for this item corresponds to 31 min or more of walking time to reach the proposed location. Generally, when the person does not know how long the journey takes, it is probably because it took longer than 31 min (38). This association justifies the equivalence of scores for each response. The total score for each item was given by the average score of its questions, except for the first item where we used the following equation: answer to the first question + (12 × answer to the second question) + (10 × answer to the third question) + (25 × fourth question-answer) + (50 × fifth question-answer) + (75 × sixth question-answer) (38). We also calculated the general score of the questionnaire, to characterize the general accessibility of the neighborhood for the practice of physical activity. For this, we used the average of the sum of the total scores of each item.

#### Use of PAL monitoring APPs

We asked participants about the use of these APPs. APPs were considered that: we're in the “Health and Fitness” category of the App Store or Play Store, that recorded the daily ANS or that estimated the caloric expenditure of physical activities performed or recorded by the user. If the participant reported the use of an APP, we registered the name of it in an open answer field of the data collection form, and later we checked the category of the APP in the smartphone store_according to the aforementioned criteria.

### Statistical analysis

Our study was divided into two parts. First, we made a profile characterization of the sample of APP users and non-users who monitor the PAL. We collected clinical, demographic, anthropometric, socioeconomic, cultural, behavioral, and psychosocial information on aspects of quality of life and the level of activity and physical fitness of all volunteers. In this step, we identifed the differences between the groups.

Subsequently, we evaluated the association between the variables raised in the characterization of the sample with the use of physical activity APPs. Those associated with the use of the APP were included in another statistical analysis, to seek independent associations between the predictor variables and the outcome variable.

First, we descriptively analyzed the data. We presented continuous variables as mean and standard deviation or as median and interquartile range and categorical variables as frequency and percentage. We verified the distribution of data using the Kolmogorov-Smirnov test. For the comparison between the groups, the Student's *t*-test and the Chi-Square test were used, for continuous and categorical variables, respectively.

The bivariate association between the predictor variables and the use of APP was assessed by calculating the Odds Ratio and its 95% confidence intervals. The predictor variables that were associated with the outcome variable with a *p*-value < 0.10 in the univariate analysis were included in a multivariate backward stepwise logistic regression analysis using likelihood ratio as a criterion to select the main predictor. Because of our enough sample, we included the following 16 independent variables: age, sex, hypertension, diabetes, dyslipidemia, smoking, schooling, peak VO_2_, socioeconomic status, fat body mass, handgrip strength, built environment, perceived stress, leg muscle strength, moderate-to-vigorous physical activity, and quality of life. We checked multicollinearity through the variation inflation factor (VIF). We found no collinearity among the above-mentioned independent variables. The conservative value of *p* < 0.10 was used considering the possibility of identifying variables with a suppressive effect, i.e., those that do not present a significant association in the univariate analysis; however, they become significant predictors in the multivariate model. This aimed to identify independent associations between the selected predictor variables and the outcome variable. We fit all models by the predictor variables described above.

We used SPSS software, version 23 (SPSS Inc., Chicago, IL, USA) for statistical analysis. The significance level adopted was 5% for all tests.

## Results

**Figure 1:**
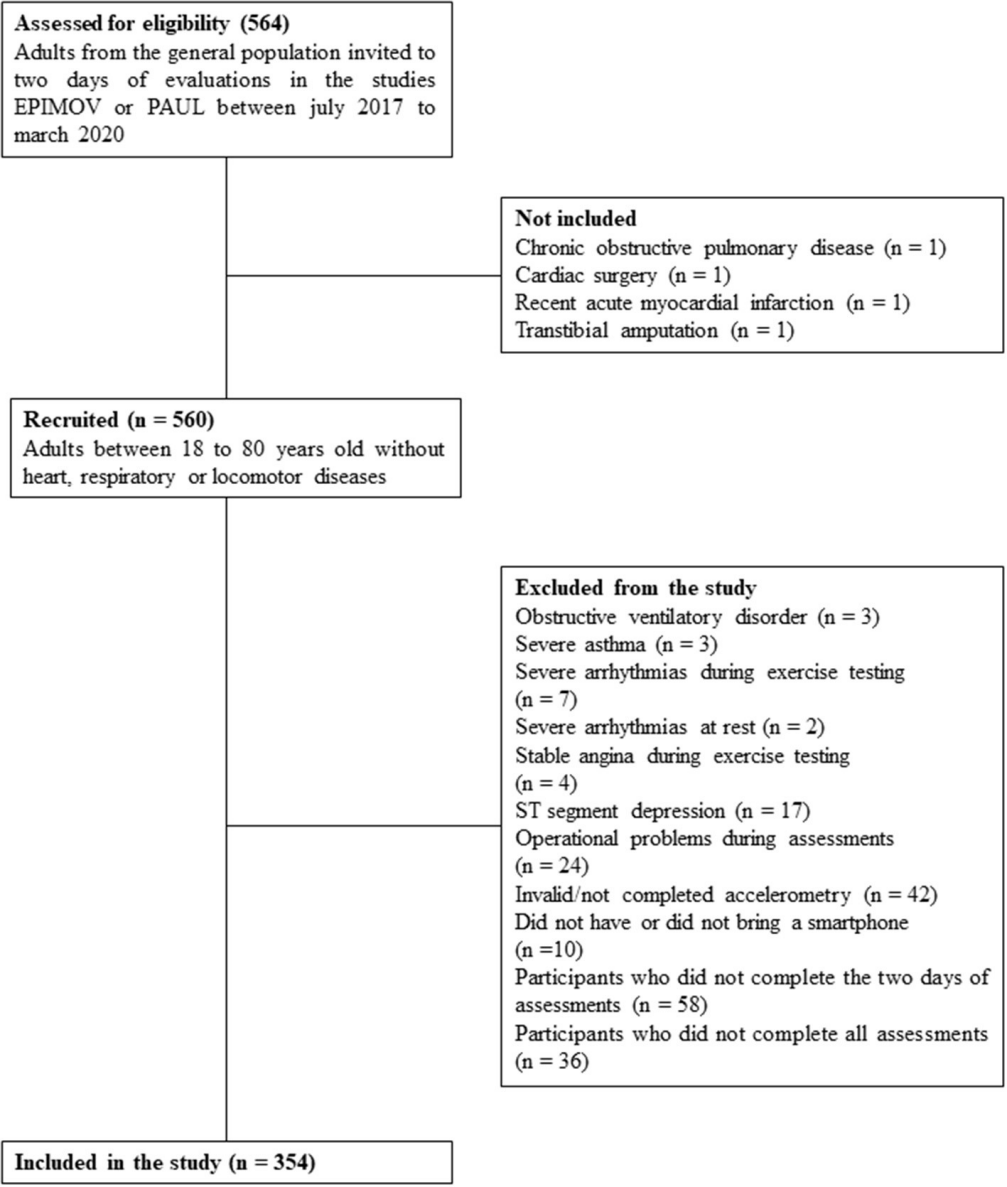
Flowchart describing the steps of inclusion and exclusion of study participants.

A total of 354 participants were evaluated and included in the statistical analyses. In Figure 1 we observe the steps of inclusion of volunteers in the present study and their respective reasons for exclusion.

Table 1 presents the general characteristics of the sample. We observed sociodemographic and anthropometric data and the presence of risk factors for the development of cardiovascular diseases.

**Table 1:** General characteristics of the studied sample.

	**Total**	**Women**	**Men**
	**(*n* = 354)**	**(*n* = 178)**	**(*n* = 176)**
Age (years)*	42 ± 12	44 ± 13	41 ± 11
**Schooling (%)**
Complete high school	59.4	59.7	59.4
Incomplete high school	40.6	40.3	40.6
**Race (%)**
White	53.9	53.2	54.4
Black	15.5	13.3	17.2
Brown	27.5	30.1	24.9
Native Indian	1.4	1.7	1.2
Asiatic	0.6	1.7	1.2
Body mass (kg)*	76 ± 17	70 ± 16	81 ± 11
Height (cm)*	166 ± 10	160 ± 6	173 ± 7
Body mass index (kg/m^2^)	27.2 ± 5.4	27.4 ± 6.1	27.1 ± 4.7
**Cardiovascular risk (%)**
Arterial hypertension	16.4	16.9	16.2
Diabetes mellitus	4.8	6.7	2.9
Dyslipidemia*	16.1	21.9	9.8
Obesity*	24.9	30.3	19.2
Current smoking	5.1	4.5	5.2
Physical inactivity*	28.0	32.6	23.1

^*^p < 0.05: men vs. women.

Our sample was predominantly white, had completed high school, was classified as overweight according to BMI, and had physical inactivity as the most prevalent risk factor for the development of cardiovascular diseases. Women had a higher prevalence of dyslipidemia, obesity, and physical inactivity when compared to men.

Table 2 presents the characteristics of the sample mentioned in the previous table, added to the data on fitness and physical activity, and the scores of the questionnaires. Here we stratified the groups between APP users and non-users who monitor PAL. The proportion of APP users found in our sample was 28.3%.

**Table 2:** General characteristics of the studied sample stratified according to the use of smartphone applications to monitor daily physical activity.

	**Users**	**Non users**
	**(*n* = 102)**	**(*n* = 252)**
Age (years)*	39 ± 10	44 ± 13
**Sex (%)**
Men	48.2	52.0
Women	51.8	48.0
**Schooling (%)**
Complete high school	69.0	55.5
Incomplete high school	31.0	44.5
**Race (%)**
White	53.9	54.0
Black	13.9	18.0
Brown	28.6	25.0
Native Indian	1.2	2.0
Asiatic	0.8	0.0
Body mass (kg)	76 ± 17	74 ± 17
Height (cm)*	166 ± 10	168 ± 8
Body mass index (kg/m^2^)*	27.7 ± 5.6	26.1 ± 4.9
Body fat (%)*	26.1 ± 7.5	30.1 + 21
Lean body mass (kg)	54 ± 12	53 ± 12
**Cardiovascular risk (%)**
Arterial hypertension	9.8	19.0
Diabetes mellitus	0.0	6.7
Dyslipidemia	9.8	18.7
Obesity	13.7	29.5
Current smoking	4.9	5.2
Physical inactivity	18.6	31.7
**Behavior stage—PA (%)**
Maintenance	63.0	47.8
Action	9.8	7.8
Preparation	7.6	28.3
Pre-contemplation	2.1	3.5
Contemplation	17.4	12.6
Socioeconomic status score*	28 ± 10	25 ±10
Quality of life score*	3.9 ± 0.5	3.8 ± 0.5
Perceived stress score	22 ± 7	23 ± 8
Built environment score	3.0 ± 0.3	2.9 ± 0.3
VO_2_ max (ml/kg/min^−1^)*	39 ± 9	32 ± 11
Peak torque KE (Nm)*	165 ± 47	147 ± 55
Grip strength (kgf)	37 ± 10	36 ± 10
Weekly MVPA (min)	341 ± 193	284 ± 232
Average number of weekly steps*	8,806 ± 3,843	7,767 ± 3,853

^*^p < 0.05: users vs. non user; PA, physical activity; KE, knee extension; MVPA, moderate to vigorous physical activity.

APP users are mostly white, with complete high school and an equivalent proportion in the distribution of the sample between the sexes. The most prevalent risk factor for cardiovascular diseases was physical inactivity, however, the time of weekly MVPA assessed via triaxial accelerometry is within the recommended by the WHO and most of the sample has been engaged in some regular practice for at least 3 months of physical activity. Adding the maintenance and action categories of the readiness questionnaire for physical exercise, the proportion of users of APP practitioners of exercise is 72.8%. Still, on the risk factors for cardiovascular diseases, the absence of diabetics among APP users is remarkable.

According to the socioeconomic status questionnaire score, the average monthly income of APP users is R$ 2,705. They also had a low level of perceived stress and self-reported quality of life in the regular classification. The perception of community environments related to physical activity was rated above the mean score, which ranges from 1 to 5.

Regarding the intergroup comparison, despite the BMI being higher in APP users, the percentage of body fat was lower among non-users. This demonstrates a more favorable body composition in APP users. Based on the indicators evaluated (VO_2_ max, peak torque of knee extension, and weekly ANS), the level of fitness and physical activity was higher in the group of APP users. The same was observed for socioeconomic status and self-reported quality of life. Despite the absence of statistically significant differences, all proportions of variables considered as risk factors were numerically lower in APP users.

Table 3 shows the independently evaluated predictors associated with APP use. Higher self-reported quality of life, socioeconomic status, and cardiorespiratory fitness positively influenced the use of APP.

**Table 3:** Results of multiple logistic regression analysis with the main attributes associated with the use of smartphone applications to monitor physical activity in the studied sample (*n* = 354).

					**Confidence interval**	
**Predictors**	**B**	**SE**	***P***	***Odds Ratio***	**Lower limit**	**Upper limit**
Age (years)	−0.062	0.024	0.009	0.940	0.897	0.984
Arterial hypertension	−1.770	0.926	0.046	0.170	0.028	0.946
VO_2_ max	0.036	0.022	0.049	1.137	1.010	1.182
*Critério Brasil*	0.091	0.028	0.001	1.096	1.037	1.157
WHOQOL BREF (Total)	1.180	0.550	0.032	3.256	1.107	9.574
Constant	−4.653	2.560	0.069	0.010	–	–

VO_2_ max, maximum O_2_ consumption on treadmill; Critério Brasil, socioeconomic assessment; WHOQOL BREF, total score of the World Health Organization quality of life questionnaire; SE, standard error.

Being hypertensive and older reduced the chance of using the APP. Partially contradicting our hypothesis, the built environment and the level of perceived stress did not present an independent association with the use of APP.

## Discussion

In the present study, we characterized the sociodemographic, clinical, health-related physical fitness profile, physical activity, and variables related to the quality of life and perception of the built environment for the practice of physical activity of APP users who monitor PAL in a sample of adults living in *Baixada Santista* region. To our knowledge, this is the first time that this public has been investigated in a Brazilian sample and compared with a group of non-APP users. This is important because previous studies evaluated residents of countries with higher socioeconomic development indices than ours (1, 4, 40).

Another important aspect was to evaluate variables that go beyond sociodemographic information, which is what we commonly find in previous studies (1, 4, 41). This makes the characterization of the APP user profile more informative and reveals some important aspects. APP users are younger and have better socioeconomic status, a fact that was already expected according to what is described in the literature (1, 4, 42). A better socioeconomic condition reflects a greater purchasing power. This facilitates access to up-to-date smartphones and an internet connection, crucial factors for obtaining and using APPs (43, 44). Age also influences a greater interest and adherence to new technologies. Older people tend to have visual difficulties, resistance to adopting new technologies, and difficulty using smartphones and their features (45). This helps us understand the difference between groups in this variable.

We observed that APP users have better physical fitness related to health and PAL. This is due to the different values obtained between groups in the assessment of body fat percentage, peak knee extension torque, VO_2_ max, and the number of weekly steps. It is important to emphasize that, although the BMI in the APP users group was higher, this does not necessarily mean a less favorable body composition. As BMI only takes into account height and body mass, it does not assess body composition (46). Therefore, the fat percentage is a more interesting variable for this comparison.

Due to the design of our study, it is not possible to establish a cause-and-effect relationship between the use of APP and the improvement of the health-related physical fitness components mentioned above. However, this draws attention to a possible demand for this type of technology by an audience that has a lower risk of developing diseases related to insufficient levels of fitness and/or physical activity. Considering that APPs are gaining strength as an emerging strategy to increase PAL, attracting new users with a profile opposite to that found in our study would be important. They are precisely the ones with the greatest potential to improve health with the use of APP.

Another factor that showed a difference in the intergroup comparison was the self-reported quality of life. This indicator was higher in the group of APP users, exposed by the higher average score obtained in the WHOQOL BREF questionnaire. The concept of quality of life evaluated involves aspects related to the perception of physical capacity, social relationships, environment, and psychological state (24). We observed that the group of APP users has higher levels of fitness and physical activity when compared to non-users. This can directly impact the quality of life of all domains evaluated, especially the physical (47). The same applies to socioeconomic status, which was also higher in APP users. Better purchasing power can directly influence the perception of all aspects of quality of life, enabling access to better health care, means of transport, security, housing, education, and leisure (48).

Contrary to what we hypothesized, there were no differences in the assessment of the built environment between the groups. We believe that factors such as better perception of safety and ease of movement on public roads could be more present in APP users, making them feel more comfortable doing physical activity on the streets carrying their smartphones. Previous results obtained by our group, during the feasibility study of developing a new APP to monitor and encourage the increase in PAL in Dutch adults, pointed to factors such as insecurity and lack of public lighting as potential barriers to its use (49). However, the built environment was not associated with the practice of low-intensity physical activity and the achievement of physical activity practice goals in a weight loss intervention (50, 51). This indicates that these potential barriers did not influence the practice of physical activity in these groups, considering that part of the increase in PAL may occur through commuting on public roads. Given this scenario of conflicting results, a more refined investigation of the components of the built environment in the behavior of APP use for PAL monitoring is interesting in future studies.

In addition to evaluating the profile of smartphone APP users who monitor PAL, we also detected variables that have an independent association with their use. The selected variables were age, arterial hypertension, VO_2_ max, socioeconomic status, and quality of life.

Each year of age tends to reduce the chance of using APP by 6%. As previously mentioned, previous studies show that APP users are younger than non-users. This difference was observed in countries such as China, United States, United Kingdom and Germany (1, 4, 15, 52). According to 2019 data from the Brazilian Institute of Geography and Statistics (IBGE), internet use tends to decrease with advancing age in Brazil (53). In the age group from 14 to 39 years old, the percentage of internet users in the population is over 90%. For the age groups of 40 to 49, 50 to 59, and 60 years or more, internet use drops to 84.6, 74.2, and 45% respectively. This decline in the percentage of use occurs precisely from the average age group observed in our sample of APP users. It is worth mentioning that the same phenomenon occurs with the percentage of people who own a smartphone for personal use (53). With the internet and the use of smartphones being necessary to access APPs, the independent association of age with APP use observed in the present study is understandable.

Moreover, a recent large study (15) evaluated the secondary source of a commercial app for physical activity to explore demographic and socioeconomic determinants of the app use. Although 77% of the app users were women, the age distribution was similar for both genders. The greatest proportion of the app users was between 25 and 50 reaching a peak proportion in people aged about 30 years old. The number of users over the age of 70 was as low as 0.5% of the sample. The average age of app users in the present study was 39, in the same age range described by Pontin et al. (15). The association between the proportion of app users and age seems to be non-linear, increasing with a peak between 35 and 44 years, reaching on average, 17.0% of weeks meeting the required physical activity guidelines, and declining in older adults regardless of gender (15).

As well as age and socioeconomic status were independently associated with APP use. Each additional point on the *Critério Brasil* questionnaire score increased the odds ratio of its use by 1.096 times. This reinforces the importance of purchasing power in the use of APPs that monitor NAF. The average income of APP users was higher than non-users in other countries (1, 4). For example, it has been reported in a developed country that app users in the highest socioeconomic strata presented on average 8,827 daily steps more than people in the lowest strata. Also, physical activity was reported as more diversified in the highest socioeconomic level (15). Also, Ernsting et al. (52) reported that health app users apart from being young, are more likely to have a college degree, report chronic conditions, and present a higher health literacy compared to non-users. This helps to understand our results. The same IBGE report mentioned above also contributes to the understanding of this event. Among the five main factors cited for not using the internet, two of them are directly related to financial factors (53). These reached 18% of the sample and were related to the costs of the internet service or smartphone acquisition. The same occurred for those who did not have a smartphone for personal use, with 27.7% of the sample claiming that the device was expensive. These data help to understand the influence of socioeconomic status on the use of APPs since current smartphones are necessary so a possible lack of processing capacity of the devices makes the use of APPs that monitor the PAL unfeasible.

The chance of using APP for PAL monitoring increased 1,137 times for each 1 ml/kg/min-1 of additional oxygen consumption. This demonstrates a positive influence of cardiorespiratory fitness on the use of APP. Litman et al. (3) investigated the level of physical activity among APP users and non-users using the IPAQ. The values of the metabolic equivalent of the task were higher both in the total score and in the vigorous domain of leisure-time physical activity in APP users. In the same sense, Shen et al. (4) reported that APP users practice more days of moderate physical activity per week. The use of APP is also common in practitioners of sports that stimulate an increase in VO_2_ max, such as running and cycling (54, 55). Considering that cardiorespiratory fitness tends to be higher in more physically active people, these results help to understand the association obtained in the present study.

Quality of life was the variable that had the highest independent association with the use of APPs that monitor PAL. Each additional point on the WHOQOL BREF questionnaire increased the odds ratio of APP use by 3.256 times. One factor that makes up the assessment of the quality of life made by this scale is the general perception of health and self-care capacity (24). These indices are positively associated with the use of APPs aimed at health (42). Another important point in assessing the quality of life is the feeling of well-being. Hershman et al. (56) reported higher levels of self-reported well-being and happiness in people who were more engaged in using APPs than in those who were less engaged. The different influences on quality of life and associated domains in the use of APP corroborate the result we found.

Finally, hypertension also influenced APP use. Being hypertensive reduced the odds ratio by 83%. The presence of comorbidities and cardiovascular risk was lower in APP users when compared to non-users in our study. These results corroborate other studies that reported a lower presence of comorbidities and chronic diseases in health and fitness APP users (4, 42). As hypertension is a chronic disease and also a cardiovascular risk factor, this helps to understand its association with the lower use of APPs that monitor PAL. Another point that contributes to the understanding of this result is the profile of hypertensive patients in Brazil. Factors such as older age, lower education, and income are more prevalent in hypertensive people (57). Considering that these variables are associated with lower use of APPs that monitor PAL, it is likely that they will have the same effect on this public.

The present study has practical applications that can facilitate a more comprehensive use of APPs that monitor PAL by the Brazilian population. First, we identified that the profile of APP users is superior in terms of fitness and PAL. This shows that the public that can benefit the most from the use of this technology is precisely the one that does it the least. Programmers can add new features to existing APPs as well as create new software that is more attractive to this consumer profile. These updates should take into account the older audience. More visible letters, simpler and easier-to-use screens, and more viable exercise suggestions for this public can facilitate their adherence to this type of technology (45).

Considering that non-users of the APP have lower financial conditions when compared to users, these potential updates should preferably be free of charge. Another important point is the communication and advertising of the APP and its functionalities to the consumer public. It is considered an important point in the decision to use an APP (44, 58). Marketing campaigns must be clear and objective to create interest and encourage people to consume the product. Considering that factors such as cardiorespiratory fitness, quality of life, and arterial hypertension influence the use of APPs, highlighting the potential effect of this technology in improving health can be an interesting path to be explored in these campaigns.

It is important to consider some limitations of our study. The sample evaluated is made up of residents of the *Baixada Santista* region, on the coast of the state of São Paulo, which is the federative unit with the highest production of wealth in Brazil (43). Added to the fact that volunteers were recruited for convenience, this prevents a more accurate extrapolation of the data to a representative sample of other regions of the country, especially those with a lower socioeconomic status. Despite the differences presented between the groups and the depth of our data, the design of our study does not allow us to establish a cause-and-effect relationship between the use of APP and the variables analyzed. Considering these factors, studies with representative samples from other locations in Brazil as well as randomized clinical trials investigating the effect of APP on variables related to fitness and physical activity will provide relevant information about these aspects in our population.

## Conclusion

Our results indicate that smartphone APPs to monitor physical activity are developed for younger adults with better socioeconomic status, lower cardiovascular risk, higher quality of life, and higher cardiorespiratory fitness. Greater efforts are needed to develop science-based applications for people who need this technology most, enabling greater potential to prevent undesirable health outcomes in asymptomatic adults.

## Data availability statement

The original contributions presented in the study are included in the article/supplementary material, further inquiries can be directed to the corresponding author/s.

## Ethics statement

The studies involving human participants were reviewed and approved by Ethics Committee in Research with Human Beings of the Federal University of São Paulo (UNIFESP - n° 0676/2018). The patients/participants provided their written informed consent to participate in this study.

## Author contributions

WV, VD, and TO: conceptualization and writing of original draft. WV and VD: data analysis and project coordination. WV, MS, and NP: methodology and project supervision. All authors: writing, review, and editing of original text. All authors contributed to the article and approved the submitted version.

## Funding

This work was funded by São Paulo Research Foundation (FAPESP) grant number 2016/50249-3. MS was funded by FAPESP/Coordenação de Aperfeiçoamento de Pessoal de Nível Superior - Brasil (CAPES), grant number 2018/21536-0.

## Conflict of interest

The authors declare that the research was conducted in the absence of any commercial or financial relationships that could be construed as a potential conflict of interest.

## Publisher's note

All claims expressed in this article are solely those of the authors and do not necessarily represent those of their affiliated organizations, or those of the publisher, the editors and the reviewers. Any product that may be evaluated in this article, or claim that may be made by its manufacturer, is not guaranteed or endorsed by the publisher.
